# Exploration of a Method of Distinguishing Different Nongxiang Tieguanyin Tea Grades Based on Aroma Determined by GC-MS Combined with Chemometrics

**DOI:** 10.3390/molecules24091707

**Published:** 2019-05-02

**Authors:** Wei Wang, Shan Jin, Yaling Guo

**Affiliations:** 1 College of Horticulture, Fujian Agriculture and Forestry University, Fuzhou 350002, China; ww1040491839@163.com (W.W.); 000q812072@fafu.edu.cn or jinshan0313@163.com (S.J.); 2 Key Laboratory of Tea Science of Fujian Province, Fujian Agriculture and Forestry University, Fuzhou 350002, China

**Keywords:** Nongxiang Tieguanyin, gas chromatography–mass spectrometry (GC-MS), aroma, differential variables

## Abstract

An aroma-based method for distinguishing different grades of Nongxiang Tieguanyin was explored by taking special grade (K110) and 1–4 grades (K101, K102, K103, and K104) of this tea as samples. Tea samples were analyzed by gas chromatography–mass spectrometry (GC-MS) combined with chemometrics. Results showed differences in the types and relative contents of aroma components among different grades of Nongxiang Tieguanyin tea. In the principal component analysis (PCA) scoring plot, except for K102 and K103, tea samples of different grades were distributed in different regions. Components satisfying variable important for the projection (VIP) > 1 and peak areas with significant differences (*p* < 0.05) among different tea grades were screened. Finally, 18 differential variables were screened out from 143 volatiles. The clustering results of these variables were consistent with those of PCA. K102 and K103 were initially clustered into one group and then clustered with K101, K110, and K104 in turn. The clear PCA separation of these samples and uniform hierarchical cluster analysis (HCA) clustering results suggests that GC-MS coupled with chemometrics analysis is a valid and accurate approach for discriminating different grades of Nongxiang Tieguanyin. The screened differential variables could represent a difference in aroma quality among five grades of Nongxiang Tieguanyin tea. Clear rules between peak area and the grade were also observed in some differential variables. 1-Ethylpyrrole and unknown-32 were positively correlated with grade. 2-Methylfuran, 2-ethylfuran, 2-methylidenecyclopentan-1-ol, mesityl oxide, 2-amylfuran, and D-limonene were negatively correlated with grade. The peak areas of methyl acetate, dimethyl sulfide, 6-methylhept-5-en-2-one, and (Z)-β-ocimene initially decreased but then increased with declining grade. The toluene content was especially high in K104 but only a negligible difference was observed among other grades. This study provides a potential method for differentiating Nongxiang Tieguanyin teas of different grades based on aroma. Unknown samples could be classified by comparison of their spatial distribution with those of known standard samples in PCA or HCA, as well as the peak area differences of differential variables between unknown samples and known standard samples.

## 1. Introduction

China is the hometown of oolong tea. Tieguanyin, the representative oolong tea in South Fujian, is mainly produced in Anxi County, Fujian Province. In the past six years, trade in the tea industry has become increasingly active, leading to an increase in Anxi Tieguanyin’s trading volume. According to statistics, in 2016, Tieguanyin’s export volume reached 10,000 t, accounting for 50% of the total export of oolong tea in the international export trade [1]. According to the National Standard GB/T 30357.2-2013 [2] approved by the Standardization Administration of China on 21 January 2016, the three types of Tieguanyin can be classified according to the different baking methods adopted in the refining of primary tea, including Qingxiang Tieguanyin, Nongxiang Tieguanyin, and Chenxiang Tieguanyin. Nongxiang Tieguanyin is the first of the three Tieguanyin types to enter the market and is processed by the most traditional method. Unique processing craftsmanship endows Nongxiang Tieguanyin with a mellow taste, long-lasting aroma, and sweet aftertaste, so it has a large share in the consumer market of Tieguanyin. The flavor of “fire” and “honey” makes it distinctly different from Qingxiang Tieguanyin and Chenxiang Tieguanyin [3].

Nongxiang Tieguanyin is usually classified into special grade and grades 1–4. The market prices of different grades vary widely. Illegal businessmen may sell seconds at best-quality prices to make profits. Inexperienced consumers may be unable to distinguish the different grades of Nongxiang Tieguanyin and misled into purchasing unnecessarily expensive tea. These issues can cause serious economic losses to the tea industry.

Appearance, aroma, taste, liquor color, and leaf bottom determine tea quality and classification [4]. Among these criteria, the proportion of aroma in the sensory evaluation of oolong tea is as high as 30%. The parameter is one of the most important traits reflecting oolong tea’s quality and it is considered by consumers before they purchase tea [5]. As the aromas of different grades of Nongxiang Tieguanyin vary greatly, tea can be classified primarily based on aroma. Sensory evaluation is the most common method of evaluating tea, but this method requires rich tea knowledge and experience. It also presents a certain degree of subjectivity. For example, aroma-based evaluation can produce different results based on changes in the evaluator’s physical and emotional condition and it is easily influenced by external factors [6]. What is more, sensory evaluation is time-consuming and inefficient. It is an inaccurate representation of the actual preferences of the public because it is done according to the personal preferences and experiences of only a few tea reviewers. These limitations make the review results difficult to quantify and digitize [7,8].

The influence of subjective factors can be eliminated by instrumental analysis simulating or assisting sensory evaluation. Gas chromatography–mass spectrometry (GC-MS) features high separation efficiency, strong identification ability, strong anti-interference ability, ease of determining the nature of the materials tested, and ability to analyze the detailed information of aroma compounds [8]. Hundreds of volatile compounds have been isolated and identified from tea by GC-MS, and the results provide a basis for the study of tea aroma compounds. GC-MS was recently used to analyze jujube [9], ginseng [10], Zhongning Goji [11], and beef [12]. GC-MS has also been applied to tea aroma analysis [13,14]. However, little is known about the difference of aroma compounds among different grades of Nongxinag Tieguanyin tea, as well as the relationship between aroma compounds and tea grades. 

Given a large number of samples analyzed by GC-MS, the amount of output data can be tremendous, and analyzing these data can be a tedious and time-consuming task. However, the brain may overlook some important datasets and potential links between datasets and result, while thinking highly of some interference datasets. Therefore, advanced chemometric methods have been developed to analyze large and complex datasets. Chemometric methods provide accurate and significant results in the quick-time domain [15]. Furthermore, it can extract relevant information and discover patterns in large series of data [16]. The most frequently used chemometric methods, including PCA [17], PLS–DA [13], and HCA [17], have been confirmed to be efficient and rapid methods to highlight differences among tea samples [18,19,20]. PCA is based on the concept of dimensionality reduction to transform multiple indicators into a few comprehensive ones, thereby simplifying the analysis process [21,22,23]. In PCA, scoring plots show the similarities and differences of various samples based on volatile component’s style and content [24]. PLS–DA is a steady discriminant statistical method that is especially suitable for cases with large numbers of explanatory variables, multicollinearity, small numbers of sample observations, and large interference noise [15,25]. VIP of PLS–DA can quantify the contribution of each variable to the classification. The greater the VIP, the more significant the difference of the variable between different grades of Nongxinag Tieguanyin tea [26]. HCA is an exploratory technique used to group samples according to the types of similarity measures used. Samples with similar spectral signatures are most likely to form a cluster [15]. Therefore, GC-MS combined with chemometric analysis may be an efficient and valid means to investigate differential aroma compounds among tea samples.

In this study, five different grades of Nongxiang Tieguanyin tea were analyzed by GC-MS coupled with chemometrics. The types and contents of aroma compounds in different grades of the tea were compared. The classification results of tea samples were observed and differential variables determining the classification of different grades of tea were screened out for the purpose of introducing the potential of an aroma-based method for differentiating Nongxiang Tieguanyin grades.

## 2. Results

### 2.1. Identification and Relative Quantification of Differential Volatile Compounds

#### 2.1.1. Identification of Differential Volatile Compounds

In this study, the raw data acquired by GC-MS included 143 compounds. Mass spectrometry (MS) and retention indices (RI) were combined to identify these compounds. Detailed information on compound identification is shown in the first five columns (No., Compounds, CAS, MS, and RI_(cal)_/RI_(ref)_) of Table 1. A total of 69 components were preliminarily identified (the “*” substances of the MS column in Table 1) by MS, 39 components were absolutely identified (the “**” substances of the MS column) by MS, and 52 components were absolutely identified by RI (the RI _(ref)_ ≠ “-” substances of the RI column). A total of 62 components were absolutely identified by MS combined with RI (including the “**” substances of the MS column or the RI _(ref)_ ≠ “-” substances of the RI column), and 46 components were preliminary identified (the substances marked “*” in the MS column and RI _(ref)_ = “-” in the RI column). Finally, 35 components could not be identified (the substances marked “unknown” and both CAS and MS columns were labeled as “-”). 

#### 2.1.2. Relative Quantification of Differential Volatile Compounds

The content differences of each compound among five grades of Nongxiang Tieguanyin tea were determined in terms of peak area ratio which were listed in the last column (Relative content) of Table 1. ANOVA (*p* < 0.05) with Duncan’s multiple range test as a post-hoc test was used to evaluate significant differences between any two pairs of treatments. 

K110, K101, K102, K103, and K104 respectively contained 107, 96, 93, 93, and 117 volatiles (i.e., the number of compounds that were not “0 ± 0” in the K110, K101, K102, K103, and K104 columns of Table 1). The five grades of tea samples shared 70 compounds (indicated compounds with an underline in the “compounds” column). A total of 62 compounds showed significant differences in relative content among tea samples of different grades (i.e., volatiles whose relative content marked with letters).

Among the 143 volatiles observed, 12 volatiles were only present in K110, including *cis*,*cis*-2,4-hexadiene (8), 3,4,5-trimethylcyclopent-2-en-1-one (28), leaf alcohol (29), 1-chloroheptane (49), cumene (51), 2-ethyltoluene (74), nopol (76), 1,1,6-trimethyl-1,2,3,4-tetrahydro-naphthalene (120), unknown-30 (135), unknown-31 (138), (Z)-α-bisabolene (139), and unknown-35 (143). The nine volatiles existed in K101, K102, K103, and K104 except for K110, including 2-methyl-3-pentanone (16), 2,6-dimethyl-1,5-heptadiene (34), methyl hexoate (43), (−)-perillyl alcohol (61), unknown-8 (73), unknown-13 (90), 3-isopropylidene-5-methyl-hex-4-en-2-one (94), propiophenone (96), and unknown-23 (118). A total of 26 volatiles only presented in K104, including methyl propanoate (7), unknown-1 (20), 2-methyltetrahydrofuran-3-one (23), methyl formate (26), 2,2-dichloroacetophenone (31), 1,1-dimethyl-4-methylenecyclohexane (40), unknown-3 (41), methyl furan-2-carboxylate (52), phenylacetaldehyde (70), acetophenone (77), 3,4,4-trimethyl-2-cyclohexen-1-one (79), unknown-10 (82), unknown-11 (83), linalool (85), nonanal (87), unknown-14 (92), 1,2,3,4-tetramethylbenzene (93), unknown-16 (97), 1,2,3,4-tetramethyl-6-isopropenyl-1,4-cyclohexadiene (104), unknown-20 (109), 2-methyl-2-phenyltridecan (110), unknown-21 (111), unknown-22 (112), *trans*-1-(but-2-en-1-yl)-3,4-dimethylbenzene (113), 1,3-dimethyl-2-butenylbenzene (115), and tridecane(117). The 12 volatiles existed in K110, K101, K102 and K103 except for K104, including 2-cyclopropylethanol (19), 2,5,5-trimethyl-1-hexen-3-yne (27), 1,4-decadiyne (53), 1-octen-3-ol (54), α-terpinene (64), unknown-12 (84), hotrienol (86), 1,3,5,8-undecatetraene (100), indole (116), α-ionone (127), unknown-26 (128), and β-ionone (133). Seven volatiles only presented in K110 and K104, including unknown-7 (55), unknown-15 (95), 6-methyl-1,3-diisopropenylcyclohexene (106), (−)-α-cedrene (125), unknown-27 (129), unknown-29 (132), and α-curcumene (134). Capronitrile (33) was only present in K101. Two volatiles existed in K110, K102, K103 and K104 except for K101, including unknown-5 (45) and unknown-34 (142). 

The relationship between relative content and grade showed obvious rules in some volatiles. The relative content of dimethyl sulfide (4) in K110 was significantly higher than that in the other tea samples, but no significant difference was observed among the remaining four grades of tea. The relative content of verbenol (78) in K110 was significantly lower than that in the other tea samples, but no significant difference was observed among the remaining four grades of tea. The relative contents of hexanal (22), 2,2,4,6,6-pentamethylheptane (57), decane (60), 2,2,4,4-tetramethyloctane (66), and dodecane (102) in K104 were significantly higher than that in the other tea samples, but no significant difference was observed among the remaining four grades of tea. The relative contents of unknown-24 (121) in K104 were significantly lower than that in the other tea samples, but no significant difference was observed among the remaining four grades of tea. The relative contents of some components, including 1-ethylpyrrole (24), 2,3,4-trimethyl-1H-pyrrole (38), 6-methylhept-5-en-2-one (56), 1,2,4-trimethylidenecyclohexane (71), indole (116), γ-muurolene (130), and unknown-32 (140), were positively correlated with tea grade. In contrast, the relative contents of some components, including 2-methyl-3-octyne (30), 2,6-dimethyl-1,5-heptadiene (34), unknown-2 (35), (+)-camphene (47), unknown-6 (48),1-octen-3-ol (54), 2-amylfuran (58), *trans*-3,5-dimethyl-1,6-octadiene (59), (−)-perillyl alcohol (61), 3-carene (62), 1-ethyl-3-methyl- benzene (63), o-cymene (65), D-limonene (67), unknown-9 (81), unknown-12 (84), propiophenone (96), 3-methylundecane (98), ionone (114), and β-ionone (133), were negatively correlated with tea grade. 

### 2.2. PCA

**Figure 1:**
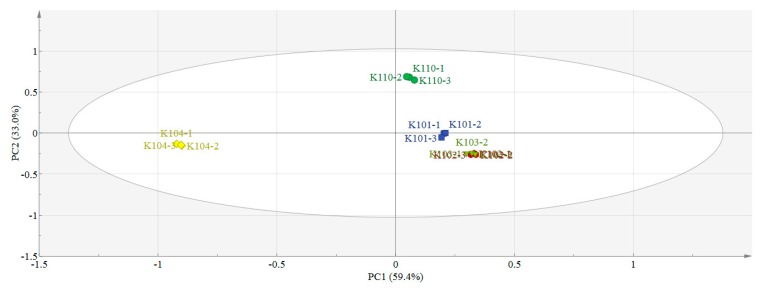
143 variables-based principal component analysis (PCA) of 5grades Nongxiang Tieguanyin: PCA score plot of principal component 1 (PC1) versus principal component 2 (PC2) scores. The tea samples are located at the distinct positions in two-dimensional space described by two vectors of PC1 = 59.4% and PC2 = 33.0%.

Visualization of the aroma properties of five different grades of Nongxiang Tieguanyin tea is presented in Figure 1 based on the 143 components in Table 1. In the PCA score plot, each “sample point” represents a test sample with three repetitions of the same color. The distance between the “sample point” and origin represents the degree, which is interpreted by principle compound (PC) 1 and 2. The more similar the aroma quality, the closer the tea sample distribution.

The first two principal components (PC1 and PC2) accounted for 92.4% of the total variance with the highest variation of 59.4% and 33.0%, respectively. The total contribution of PC1 and PC2 to the variance was over 90%, which indicates that the first two PCs are sufficient to explain the total variance of the dataset [27].

The results of the three repeats of each tea sample were aggregated. K110, K104, and the three other tea samples were in the first, third, and fourth quadrants, respectively. In terms of grade, K110, K101, K102, and K103 were on the right side of the Y-axis, whereas K104 was on the left side of this axis. The clear separation of K104 from K110 was based on PC1 and PC2 while that of K104 from K101, K102, and K103 was mainly based on PC1. This result indicates that the aroma quality of K104 is quite different from that of the other samples. K102 and K103 overlapped markedly, which suggests that they have similar aroma quality. However, K110, K101, and K102 or K110, K101, and K103 differed from one another based on PC1 and PC2. K110, K101, K102 and K103 samples are successively located from the upper left of the Y-axis to the lower right of the Y-axis in the PCA spatial domain, their ranking order coincides with the order of their grades, which gradually decrease from K110 to K103. The results showed that different grades of Nongxiang Tieguanyin tea can be graded by GC-MS combined with chemometric methods.

### 2.3. PLS–DA

Not all 143 compounds in Table 1 play an important role in the differentiation of the five grades of tea samples. To eliminate the interference of irrelevant variables and find the key compounds that affect the classification of tea samples, PLS–DA was used to screen the components with VIP > 1. Then, ANOVA was used to further screen compounds with significant differences in peak areas among different grades of tea samples (*p* < 0.05). Finally, 18 volatiles were screened out and considered as differential variables (Table 2).

**Figure 2:**
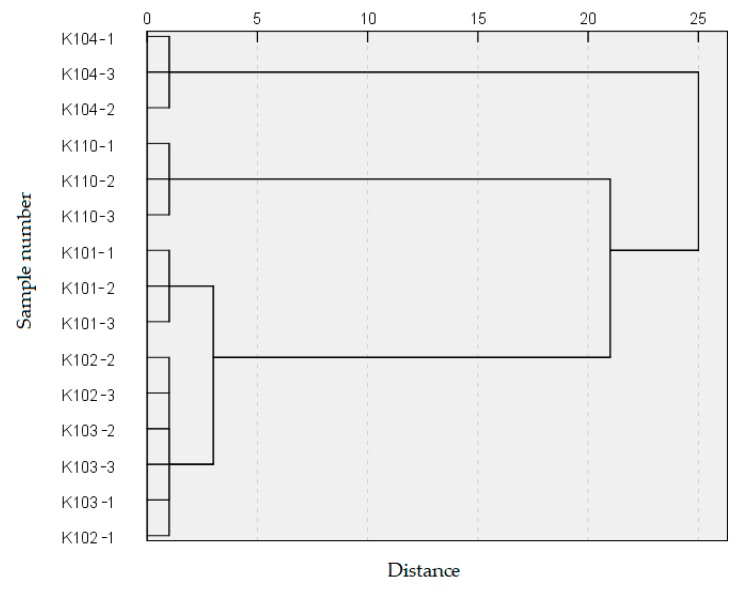
Unsupervised hierarchical clustering of Nongxiang Tieguanyin based on the relative content of 18 differential variables. Three technical replicates were represented for each sample with “-1”, “-2” and “-3” following the sample number.

To test whether the selected 18 differential variables could distinguish different grades, HCA of the five grades tea samples was carried out with the relative content of the differential variables. As shown in Figure 2, a dendrogram is the mapping result of the evaluation index of the tested object according to the unified scale. When the dendrogram was cut with the vertical line at the distance 5, the tea samples of five grades could be classified into three categories, including K104 in a category, K110 in a second category, and grades 1–3 (K101, K102, and K103) in a third category.

In summary, K102 and K103 were initially clustered into one group and clustered with K101, K110, and K104 in turn. In other words, tea samples with similar aroma quality are preferentially clustered into one group. These results are in agreement with those obtained by PCA based on 143 volatiles, thus indicating that the 18 differential variables could represent differences in aroma quality among five grades of Nongxiang Tieguanyin tea. 

As shown in Table 2, clear rules between the peak area and the grade were also observed in some differential variables. Two compounds, including 1-ethylpyrrole (24) and unknown-32 (140), were positively correlated with grade, whereas six compounds, namely, 2-methylfuran (5), 2-ethylfuran (12), 2-methylidenecyclopentan-1-ol (17), mesityl oxide (21), 2-amylfuran (58), and D-limonene (67), were negatively correlated with grade. Four compounds, including methyl acetate (3), dimethyl sulfide (4), 6-methylhept-5-en-2-one (56), and (Z)-β-ocimene (72), at low contents, increased the tea aroma quality; however, excess contents of these compounds decreased the tea aroma quality. The toluene (18) content of K104 was especially high, but only a negligible difference was observed among other grades. No obvious rule was established between the remaining five differential variables, including that of 2-methylbutanal (10), 1-penten-3-ol (11), unknown-4 (44), hotrienol (86), and benzyl cyanide (91), and grade.

## 3. Discussion

The GC-MS results of this study suggest that the types and contents of aroma components differ among five different grades of Nongxiang Tieguanyin tea. Some volatiles were found in only one or some grades of tea samples, and obvious rules (i.e., relative content had positive or negative correlations with grade) between the relative content and tea grade were observed in some volatiles. 

In the PCA scoring plot, tea samples of different grades, except K102 and K103, were distributed in different regions. However, not all 143 volatiles detected by GC-MS played an important role in the differentiation of the five grades of tea samples. A total of 18 differential variables were selected by PLS–DA combined with ANOVA. Clear separation of five samples in PCA and uniform clustering results in HCA suggests that GC-MS coupled with chemometric analysis is a valid and accurate approach to discriminate different grades of Nongxiang Tieguanyin tea. Moreover, the 18 differential variables were adequate to represent differences in aroma among different grades of Nongxiang Tieguanyin, so they could be used as grading criteria. In other words, they may serve as a reference for identifying the grade to which an unknown sample belongs. Some differential variables, such as 1-ethylpyrrole (24) and unknown-32 (140), were positively correlated with grade; in contrast, 2-methylfuran (5), 2-ethylfuran (12), 2-methylidenecyclopentan-1-ol (17), mesityl oxide (21), 2-amylfuran (58), and D-limonene (67) were negatively correlated with grade.

This study introduced a potential method for differentiating Nongxiang Tieguanyin tea of different grades based on aroma. If the unknown samples are analyzed by GC-MS according to the same method applied in this study, and the results are analyzed by PCA. According to the spatial distributions of the PCA score plot, the unknown samples could be preliminarily identified. The peak areas of 18 differential variables detected in unknown samples will be compared with those of five standard samples (K110, K101, K102, K103, and K104) to enable grade identification of an unknown sample. For example, if the unknown sample is close to K104 in the PCA score plot and its’ toluene peak area is very high, the unknown sample may be grade 4. If the unknown sample is close to K110 in the PCA score plot, and the peak areas of 1-ethylpyrrole (24) and unknown-32 (140) are significantly higher than that of other tea samples, whereas the peak areas of 2-methylfuran (5), 2-ethylfuran (12), 2-methylidenecyclopentan-1-ol (17), mesityl oxide (21), 2-amylfuran (58), and D-limonene (67) are significantly lower than other tea samples (i.e., the peak areas of differential variables in unknown sample are close to K110), the unknown sample should be special grade. It is noticeable that there are two unknown compounds in the screened differential variables. Among them, unknown-4 was not correlated with the grade, while unknown-32 were positively correlated with the grade. Due to the matching degrees of unknown compounds were lower than 700, we defined them as “unknown” in this article. However, the measured retention indexes of them were fixed. So even if the unknown compound could not be identified, the peak area of it in different samples can be compared only if unknown tea samples are analyzed under the same experimental conditions of GC-MS as the known tea samples and the unknown compound in different samples have similar retention index. In addition, to determine the grade of unknown tea samples, the peak area of correlative differential variables should be taken into account comprehensively, instead of only consider the peak area of one or two differential variables among them. Next, we will also look for more methods to identify the two unknown compounds.

Note that this study only explored the proposed method without further validation. If the selected differential variable’s peak area of an unknown sample is located between two adjacent grades, or if some differential variables are not detected in the unknown sample, accurately grading the sample may be difficult or impossible. Therefore, in future studies, large experimental samples should be analyzed in order to promote the reliability and practicability of this method in distinguishing different Nongxiang Tieguanyin grades.

## 4. Materials and Methods

### 4.1. Tea Samples, Reagents and Instruments

Special grade and grade 1–4 of Anxi Nongxiang Tieguanyin tea made in autumn of 2017 were selected as samples in the study (Table 3) [2]. After being ground and poured into a 40-mesh sieve, the tea powder, which had passed through the sieve, was collected and put in aluminum foil bags in order to be sealed for reserving.

NaCl (analytically pure reagent, purity ≥ 99.5%) was purchased from Shanghai Guo Yao Group Chemical Reagent limited company (Shanghai, China). Deionized water from a water purification system (Kertone water treatment Co., Ltd., Changsha, China) was used in all experiments. A standard mixture of n-alkanes C8-C30 was purchased from o2si (South Carolina, American).

The GC-MS system consisted of Clarus SQ8T GC-MS, Elite-FFAP chromatographic column of which the specifications were 30 m × 0.25 mm × 0.25 μm and TurboMatrix 40 Trap automatic headspace sampler (Perkin Elmer, Waltham, Massachusetts, American). In addition, electronic analytical balance (precision run up to 0.0001 g, Mettle Toledo instruments Co., Ltd., Shanghai, China), WK-400B high speed pharmaceutical pulverizer (Shandong Qingzhou Jingcheng Machinery Co., Ltd., Qingzhou, China), 40 mesh standard inspection sieve (Shangyu Yinhe testing instrument factory, Shangyu, China) were used in this experiment.

### 4.2. HS/GC-MS Method

#### 4.2.1. Headspace Injection Method for Tea Sample

Tea powder (0.5000 g) and 1 mL saturated NaCl (0.28 g/mL) which was dissolved in the deionized water were placed into the 20 mL headspace bottle. Then the bottles were sealed with a hermetical cap equipped with polytetrafluoroethylene (PTFE)/silicone white septa stopper and placed in the automatic sampler in order. Finally, tea samples in the automatic sampler were sampled with ready-set instrument parameters. Each tea sample was repeatedly tested three times.

Equilibrium temperature was 80 °C, sampling needle temperature was 100 °C, transmission line temperature was 120 °C, equilibrium time was 60 min, highest and lowest temperature of collecting trap were 280 °C and 40 °C, respectively. The samples were kept for 5 min, blown for 1 min, desorbed for 0.5 min. The headspace bottle pressure was 40 psi, chromatographic column pressure was 12 psi, desorption pressure was 15 psi. The headspace outlet was shunted. 

#### 4.2.2. GC-MS Condition

The heating program of oven temperature was as follows: initial temperature was 50 °C (held for 5 min), next it was heated to 125 °C at the rate of 3 °C/min (held for 2 min), then it was heated to 180 °C at the rate of 5 °C/min (held for 3 min), finally it was heated to 230 °C at the rate of 15 °C/min (held for 5 min). Helium (purity was 99.999%) was used as the carrier gas. The MS fragmentation was performed by electronic impact (EI) mode whose ionization energy was 70 eV and the source temperature was 230 °C. The MS transmission line temperature was 250 °C. The acquisition was carried out in full-scan mode in the range of 45–500 *m*/*z*.

#### 4.2.3. Headspace Sampling Method and Retention Index Calculation of N-Alkanes

Two microliter of standard mixture of n-alkanes was mensurated according to HS and GC-MS parameters in accordance with tea samples and repeated three times. The retention time of each n-alkanes was recorded three times and the average value was calculated. The retention index of each component was calculated by Kovats retention index formula [28]:

(1) $$\mathrm{RI}=100n+100*(\frac{{\mathrm{RT}}_{\mathrm{unknown}}{-\mathrm{RT}}_{n}}{{\mathrm{RT}}_{n+1}{-\mathrm{RT}}_{n}})$$

Note: RI—retention index of components to be measured; RT_unknown_—retention time of components to be measured; RT_n_, RT_n+1_—retention time of n-alkanes before and after the components to be measured.

### 4.3. Analysis

The raw data acquired by GC-MS was first identified: (1) by comparing their mass spectra with information from NIST 11 library (whether matching score > 700); (2) by comparing their measured retention index (RI) with the theoretical RI provided by previous literature [29,30,31,32]. 

Next, the quantitative identification was determined by peak area normalization method, i.e., the relative content was expressed by the ratio of the peak area of each aroma component to the total peak area. Data were expressed as mean ± SD (standard deviation).

Then, these data were submitted to PCA, PLS-DA, ANOVA and HCA, a series of statistical and clustering analysis. PCA and PLS-DA were performed using the SIMCA v14.1 (Soft Independent Modelling by Class Analogy, umetrics, Sweden), a chemometric software for data filtering. PCA was used to verify the effect of the instrument in distinguishing different grades of Nongxiang Tieguanyin tea. PLS-DA was used to screen the components of VIP > 1. ANOVA and HCA were performed by SPSS v21.0 (SPSS Inc., Chicago, IL, USA). ANOVA was used to investigate the differences of aroma compounds’ relative content and peak area among the five grades of tea samples. ANOVA was applied with a *p*-value < 0.05. HCA was used to verify the discriminative effect of differential variables on tea samples.

## 5. Conclusions

In this study, different grades of Nongxiang Tieguanyin tea were analyzed by GC-MS. The types and contents of volatiles in five tea grades varied. In PCA, tea samples of different grades were distributed in different quadrants of an orthogonal two-dimensional coordinate system, except for K102 and K103, which overlapped markedly. Following the criteria of VIP > 1 in PLS–DA and *p* < 0.05 in ANOVA, 18 differential variables affecting the classification of tea samples were screened. The clustering results based on 18 differential variables of five tea grades were consistent with the PCA. Clear separation of these samples in PCA and uniform clustering results in HCA suggest that GC-MS coupled with chemometric analysis is a valid and accurate approach to discriminate different grades of Nongxiang Tieguanyin tea. Obvious rules between the peak area and grade were observed in some differential variables. For example, 1-ethylpyrrole and unknown-32 were positively correlated with tea grade, but 2-methylfuran, 2-ethylfuran, 2-methylidenecyclopentan-1-ol, mesityl oxide, 2-amylfuran, and D-limonene were negatively correlated with tea grade. The peak areas of methyl acetate, dimethyl sulfide, 6-methylhept-5-en-2-one, and (Z)-β-ocimene initially decreased but then increased with decreasing grade. The toluene content of K104 was especially high but showed little difference among the four other grades. Therefore, the 18 differential variables identified in this work can be used as indicators to distinguish Nongxiang Tieguanyin tea of different grades. This information may help in the classification of unknown samples through comparison of the spatial distribution of unknown samples with that of known standard samples in PCA or HCA and calculation of the peak area of differential variables.

## Figures and Tables

**Table 1:** The qualitative and quantitative results of five grades tea samples.

No.	Compounds	CAS ^a^	MS ^b^	RI_(cal)_/RI_(ref)_^c^	Relative Content (Mean ± SD)/% ^d^				
					K110	K101	K102	K103	K104
1	methyl chloride	74-87-3	**	-/-	2.73 ± 0.1a	1.76 ± 0.26c	2.88 ± 0.22a	2.35 ± 0.08b	0.92 ± 0.06d
2	oxetane	503-30-0	*	-/-	3.01 ± 0.16abc	3.13 ± 0.15ab	2.78 ± 0.18c	2.95 ± 0.19bc	3.27 ± 0a
3	methyl acetate	79-20-9	*	-/-	11.17 ± 0.35a	7.03 ± 0.43b	5.83 ± 0.65c	5.79 ± 0.5c	11.83 ± 0.15a
4	dimethyl sulfide	75-18-3	**	-/-	11.49 ± 0.97a	7.52 ± 0.79b	7.09 ± 0.36b	7.35 ± 0.37b	8.13 ± 0.1b
5	2-methylfuran	534-22-5	*	-/604	2.84 ± 0.13c	3.33 ± 0.04b	3.66 ± 0.13a	3.4 ± 0.18ab	3.48 ± 0.17ab
6	hexane	110-54-3	**	-/-	2.42 ± 0.1bc	2.52 ± 0.13ab	2.58 ± 0.09ab	2.66 ± 0.09a	2.28 ± 0.06c
7	methyl propanoate	554-12-1	*	-/-	0 ± 0	0 ± 0	0 ± 0	0 ± 0	1.28 ± 0.21
8	cis,cis-2,4-hexadiene	6108-61-8	*	-/-	0.33 ± 0.01	0 ± 0	0 ± 0	0 ± 0	0 ± 0
9	isovaleraldehyde	590-86-3	**	-/656	0.71 ± 0b	0.69 ± 0.03b	0.85 ± 0.04a	0.88 ± 0.05a	0.48 ± 0.01c
10	2-methylbutanal	96-17-3	**	-/665	6.77 ± 0.26c	6.08 ± 0.18d	7.93 ± 0.19b	8.71 ± 0.32a	3.82 ± 0.06e
11	1-penten-3-ol	616-25-1	*	-/681	6.85 ± 0.2d	13.91 ± 0.34c	15.92 ± 0.68a	14.67 ± 0.33b	6 ± 0.12e
12	2-ethylfuran	3208-16-0	*	-/701	3.1 ± 0.09c	4.6 ± 0.28a	4.73 ± 0.13a	4.88 ± 0.28a	3.6 ± 0.34b
13	isocyanoethane	624-79-3	*	-/-	1.08 ± 0.09	1.12 ± 0.01	0.85 ± 0.02	0.88 ± 0.03	1.57 ± 0.1
14	isovaleronitrile	625-28-5	**	-/-	0.37 ± 0.01b	0.38 ± 0.04b	0.26 ± 0.01c	0.3 ± 0.02c	0.45 ± 0.03a
15	tiglic aldehyde	1115-11-3	*	-/744	0.29 ± 0.02	0.41 ± 0.05	0.35 ± 0.01	0.36 ± 0.02	0.46 ± 0.02
16	2-methyl-3-pentanone	565-69-5	*	-/-	0 ± 0	0.18 ± 0.02	0.17 ± 0.01	0.12 ± 0	0.22 ± 0.03
17	2-methylidenecyclopentan-1-ol	20461-31-8	*	-/-	1.41 ± 0.06e	2.23 ± 0.11c	2.44 ± 0.07b	2.71 ± 0.08a	1.97 ± 0.03d
18	toluene	108-88-3	**	-/773	2.3 ± 0.02b	2.3 ± 0.19b	1.93 ± 0.24c	2.04 ± 0.07bc	5.66 ± 0.12a
19	2-cyclopropylethanol	2566-44-1	*	-/-	1.38 ± 0.04	3.28 ± 0.07	3.27 ± 0.23	3.38 ± 0.21	0 ± 0
20	unknown-1	-	-	-/-	0 ± 0	0 ± 0	0 ± 0	0 ± 0	0.31 ± 0.01
21	mesityl oxide	141-79-7	*	-/-	0.61 ± 0.02c	0.75 ± 0.06b	1.2 ± 0.09a	1.19 ± 0.04a	0.71 ± 0.03bc
22	hexanal	66-25-1	**	-/801	1.03 ± 0.03b	0.95 ± 0.04b	0.94 ± 0.05b	0.93 ± 0.05b	1.31 ± 0.1a
23	2-methyltetrahydrofuran-3-one	3188-00-9	**	805/-	0 ± 0	0 ± 0	0 ± 0	0 ± 0	0.31 ± 0.02
24	1-ethylpyrrole	617-92-5	*	808/-	12.27 ± 0.7a	8.66 ± 0.08b	6.94 ± 0.43c	7.05 ± 0.01c	1.63 ± 0.09d
25	furfural	98-01-1	**	830/-	0.3 ± 0.02	0.92 ± 0.09	0.71 ± 0.05	0.55 ± 0.06	7.08 ± 0.44
26	methyl formate	107-31-3	*	835/-	0 ± 0	0 ± 0	0 ± 0	0 ± 0	4.96 ± 0.82
27	2,5,5-trimethyl-1-hexen-3-yne	37439-53-5	*	837/-	0.31 ± 0.01	0.78 ± 0.03	0.63 ± 0.05	0.84 ± 0.05	0 ± 0
28	3,4,5-trimethylcyclopent-2-en-1-one	55683-21-1	*	847/-	0.05 ± 0	0 ± 0	0 ± 0	0 ± 0	0 ± 0
29	leaf alcohol	928-96-1	*	849/853	0.11 ± 0.01	0 ± 0	0 ± 0	0 ± 0	0 ± 0
30	2-methyl-3-octyne	55402-15-8	*	852/-	0.09 ± 0c	0.37 ± 0.02b	0.33 ± 0.02b	0.45 ± 0.07a	0.44 ± 0.03a
31	2,2-dichloroacetophenone	2648-61-5	*	854/-	0 ± 0	0 ± 0	0 ± 0	0 ± 0	0.5 ± 0.02
32	p-Xylene	106-42-3	*	863/-	0.94 ± 0.01c	1.27 ± 0.02b	0.79 ± 0.04d	0.81 ± 0.06d	1.75 ± 0.06a
33	capronitrile	628-73-9	*	874/-	0 ± 0	0.07 ± 0.01	0 ± 0	0 ± 0	0 ± 0
34	2,6-dimethyl-1,5-heptadiene	6709-39-3	*	878/-	0 ± 0	0.08 ± 0.01	0.09 ± 0.01	0.15 ± 0.01	0.29 ± 0.02
35	unknown-2	-	-	887/-	0.33 ± 0.01	0.68 ± 0.05	0.67 ± 0.03	0.68 ± 0.02	0.93 ± 0.01
36	cis-4-heptenal	6728-31-0	*	898/901	0.05 ± 0c	0.11 ± 0a	0.05 ± 0.01c	0.06 ± 0.01b	0.05 ± 0.01c
37	heptanal	111-71-7	**	901/903	0.08 ± 0.01bc	0.1 ± 0.01a	0.07 ± 0.01c	0.08 ± 0.01c	0.1 ± 0.01ab
38	2,3,4-trimethyl-1H-pyrrole	3855-78-5	*	903/-	0.65 ± 0.03	0.55 ± 0.04	0.46 ± 0.01	0.42 ± 0.02	0.16 ± 0
39	1-(furan-2-yl)ethanone	1192-62-7	*	909/902	0.15 ± 0.01d	0.37 ± 0.08b	0.71 ± 0.03a	0.42 ± 0.02b	0.26 ± 0.02c
40	1,1-dimethyl-4-methylenecyclohexane	6007-96-1	*	910/-	0 ± 0	0 ± 0	0 ± 0	0 ± 0	0.29 ± 0.02
41	unknown-3	-	-	912/-	0 ± 0	0 ± 0	0 ± 0	0 ± 0	0.08 ± 0.01
42	2-amino-5-methylpyridine	1603-41-4	*	917/-	0.92 ± 0.04	1.06 ± 0.07	0.68 ± 0.02	0.65 ± 0.05	0.25 ± 0
43	methyl hexoate	106-70-7	**	922/925	0 ± 0c	0.27 ± 0.01a	0.2 ± 0.01b	0.21 ± 0.02b	0.26 ± 0.01a
44	unknown-4	-	-	928/-	0.09 ± 0cd	0.08 ± 0d	0.22 ± 0.01a	0.1 ± 0.01b	0.1 ± 0.01bc
45	unknown-5	-	-	937/-	0.03 ± 0d	0 ± 0e	0.05 ± 0c	0.06 ± 0.01b	0.11 ± 0a
46	1-butylpyrrole	589-33-3	*	940/-	0.07 ± 0a	0.06 ± 0a	0.03 ± 0b	0.07 ± 0.01a	0.03 ± 0.01b
47	(+)-camphene	79-92-5	*	943/955	0.04 ± 0d	0.05 ± 0d	0.05 ± 0c	0.06 ± 0b	0.08 ± 0.01a
48	unknown-6	-	-	952/-	0.04 ± 0d	0.05 ± 0.01c	0.05 ± 0c	0.07 ± 0b	0.21 ± 0.01a
49	1-chloroheptane	629-06-1	*	955/-	0.03 ± 0	0 ± 0	0 ± 0	0 ± 0	0 ± 0
50	benzaldehyde	100-52-7	**	957/957	0.95 ± 0.03	1.64 ± 0.2	1.42 ± 0.02	1.14 ± 0.04	3.11 ± 0.28
51	cumene	98-82-8	*	961/-	0.34 ± 0.01	0 ± 0	0 ± 0	0 ± 0	0 ± 0
52	methyl furan-2-carboxylate	611-13-2	*	971/-	0 ± 0	0 ± 0	0 ± 0	0 ± 0	0.5 ± 0.01
53	1,4-decadiyne	929-53-3	*	972/-	0.13 ± 0.01c	0.2 ± 0.02a	0.17 ± 0.01b	0.18 ± 0.01ab	0 ± 0d
54	1-octen-3-ol	3391-86-4	**	980/980	0.05 ± 0	0.08 ± 0	0.1 ± 0	0.11 ± 0	0 ± 0
55	unknown-7	-	-	981/-	0.06 ± 0	0 ± 0	0 ± 0	0 ± 0	0.15 ± 0.01
56	6-methylhept-5-en-2-one	110-93-0	**	983/982	1.58 ± 0.08a	1.63 ± 0.02a	1.4 ± 0.07b	1.37 ± 0.06b	0.85 ± 0.03c
57	2,2,4,6,6-pentamethylheptane	13475-82-6	*	986/-	1.06 ± 0.11b	1 ± 0.06b	1.04 ± 0.09b	0.94 ± 0.05b	1.84 ± 0.06a
58	2-amylfuran	3777-69-3	**	987/986	1.39 ± 0.18c	1.8 ± 0.14b	2.14 ± 0.21a	2.28 ± 0.11a	2.11 ± 0.08a
59	trans-3,5-dimethyl-1,6-octadiene	74630-87-8	*	997/-	0.19 ± 0.03d	0.27 ± 0.04c	0.33 ± 0.04b	0.38 ± 0.01b	0.47 ± 0.02a
60	decane	124-18-5	**	999/1000	0.06 ± 0.01b	0.06 ± 0.01b	0.07 ± 0.01b	0.07 ± 0.01b	0.12 ± 0.01a
61	(−)-perillyl alcohol	18457-55-1	**	1003/-	0 ± 0	0.19 ± 0.01	0.22 ± 0.04	0.29 ± 0.02	0.38 ± 0.03
62	3-carene	13466-78-9	*	1013/1014	0.09 ± 0.01c	0.14 ± 0.02b	0.19 ± 0.02a	0.19 ± 0.01a	0.21 ± 0.01a
63	1-ethyl-3-methyl- benzene	620-14-4	*	1015/-	0.13 ± 0.01d	0.18 ± 0.02c	0.21 ± 0.02b	0.21 ± 0.01b	0.29 ± 0.01a
64	α-terpinene	99-86-5	*	1018/1021	0.06 ± 0.01	0.06 ± 0.01	0.06 ± 0	0.06 ± 0.01	0 ± 0
65	o-cymene	527-84-4	**	1020/1022	0.13 ± 0.02d	0.19 ± 0.02c	0.24 ± 0.02b	0.27 ± 0.01b	1.29 ± 0.04a
66	2,2,4,4-tetramethyloctane	62183-79-3	**	1023/-	0.07 ± 0.01b	0.08 ± 0b	0.08 ± 0.01b	0.07 ± 0.01b	0.11 ± 0.01a
67	D-limonene	5989-27-5	**	1025/1025	0.46 ± 0.06d	0.75 ± 0.08c	0.98 ± 0.12b	1.09 ± 0.11b	1.25 ± 0.03a
68	2,2,6-trimethylcyclohexanone	2408-37-9	**	1031/1031	0.18 ± 0.02c	0.31 ± 0.02a	0.34 ± 0.02a	0.32 ± 0.01a	0.23 ± 0.01b
69	(E)-β-ocimene	3779-61-1	**	1034/1035	0.13 ± 0.02b	0.12 ± 0.01b	0.17 ± 0.02a	0.19 ± 0a	0.11 ± 0b
70	phenylacetaldehyde	122-78-1	**	1040/1040	0 ± 0	0 ± 0	0 ± 0	0 ± 0	0.04 ± 0.01
71	1,2,4-trimethylidenecyclohexane	14296-81-2	*	1042/-	0.45 ± 0.08a	0.33 ± 0.02b	0.24 ± 0.03b	0.35 ± 0.02c	0.11 ± 0.01d
72	(Z)-β-ocimene	13877-91-3	**	1044/1044	3.02 ± 0.26	2.69 ± 0.08	2.32 ± 0.09	2.41 ± 0.05	2.69 ± 0.04
73	unknown-8	-	-	1050/-	0 ± 0d	0.18 ± 0.01b	0.15 ± 0.02b	0.42 ± 0.02a	0.11 ± 0.01c
74	2-ethyltoluene	611-14-3	*	1052/-	0.14 ± 0.01	0 ± 0	0 ± 0	0 ± 0	0 ± 0
75	isophorone	78-59-1	*	1055/1050	0.12 ± 0e	0.27 ± 0.01c	0.33 ± 0.02b	0.48 ± 0.02a	0.24 ± 0.01d
76	nopol	128-50-7	*	1058/-	0.06 ± 0	0 ± 0	0 ± 0	0 ± 0	0 ± 0
77	acetophenone	98-86-2	*	1062/1062	0 ± 0	0 ± 0	0 ± 0	0 ± 0	0.1 ± 0.01
78	verbenol	473-67-6	*	1069/-	0.33 ± 0.03b	0.56 ± 0.04a	0.67 ± 0.08a	0.66 ± 0.04a	0.57 ± 0.1a
79	3,4,4-trimethyl-2-cyclohexen-1-one	17299-41-1	*	1075/-	0 ± 0	0 ± 0	0 ± 0	0 ± 0	0.1 ± 0.01
80	terpinolene	586-62-9	**	1081/1089	0.11 ± 0.01c	0.14 ± 0.02c	0.23 ± 0.03a	0.23 ± 0.01a	0.17 ± 0.01b
81	unknown-9	-	-	1086/-	0.13 ± 0.01d	0.2 ± 0.01c	0.29 ± 0.03ab	0.26 ± 0.03b	0.32 ± 0.02a
82	unknown-10	-	-	1091/-	0 ± 0	0 ± 0	0 ± 0	0 ± 0	0.06 ± 0.01
83	unknown-11	-	-	1094/-	0 ± 0	0 ± 0	0 ± 0	0 ± 0	0.04 ± 0.01
84	unknown-12	-	-	1099/-	0.28 ± 0.03b	0.28 ± 0.02b	0.35 ± 0.01a	0.35 ± 0.01a	0 ± 0c
85	linalool	78-70-6	*	1099/1100	0 ± 0	0 ± 0	0 ± 0	0 ± 0	0.1 ± 0
86	hotrienol	20053-88-7	*	1102/1104	1.13 ± 0.02	0.96 ± 0.07	1.88 ± 0.04	1.81 ± 0.1	0 ± 0
87	nonanal	124-19-6	**	1102/1105	0 ± 0	0 ± 0	0 ± 0	0 ± 0	0.02 ± 0
88	N-methyl-4-anisidine	5961-59-1	*	1109/1119	0.23 ± 0.01a	0.19 ± 0.01b	0.23 ± 0.01a	0.19 ± 0.02b	0.13 ± 0.02c
89	2-ethenyl-1,1-dimethyl-3-methylenecyclohexane	95452-08-7	*	1112/-	0.91 ± 0.12	0.5 ± 0.05	0.41 ± 0.07	0.4 ± 0.02	0.72 ± 0.02
90	unknown-13	-	-	1127/-	0 ± 0	0.05 ± 0.01	0.08 ± 0	0.09 ± 0.01	0.06 ± 0
91	benzyl cyanide	140-29-4	**	1136/1135	1.29 ± 0.1	1.07 ± 0.02	0.5 ± 0.03	0.51 ± 0.01	0.55 ± 0.01
92	unknown-14	-	-	1140/-	0 ± 0	0 ± 0	0 ± 0	0 ± 0	0.16 ± 0.01
93	1,2,3,4-tetramethylbenzene	488-23-3	*	1144/-	0 ± 0	0 ± 0	0 ± 0	0 ± 0	0.11 ± 0
94	3-isopropylidene-5-methyl-hex-4-en-2-one	64149-32-2	*	1149/-	0 ± 0	0.12 ± 0	0.15 ± 0.03	0.13 ± 0.02	0.06 ± 0.01
95	unknown-15	-	-	1152/-	0.09 ± 0	0 ± 0	0 ± 0	0 ± 0	0.06 ± 0.01
96	propiophenone	93-55-0	*	1161/-	0 ± 0	0.1 ± 0	0.11 ± 0.02	0.12 ± 0	0.21 ± 0.01
97	unknown-16	-	-	1164/-	0 ± 0	0 ± 0	0 ± 0	0 ± 0	0.06 ± 0.01
98	3-methylundecane	1002-43-3	**	1170/1176	0.07 ± 0b	0.07 ± 0.01b	0.09 ± 0a	0.08 ± 0.01a	0.08 ± 0.01a
99	unknown-17	-	-	1176/-	0.26 ± 0.01b	0.32 ± 0.01a	0.3 ± 0.01a	0.25 ± 0.01b	0.12 ± 0.02c
100	1,3,5,8-undecatetraene	50277-31-1	*	1186/1185	0.13 ± 0.01	0.14 ± 0.01	0.11 ± 0.02	0.13 ± 0	0 ± 0
101	safranal	116-26-7	**	1194/-	0.09 ± 0d	0.22 ± 0.01c	0.31 ± 0.01a	0.27 ± 0.01b	0.07 ± 0.01e
102	dodecane	112-40-3	**	1200/1200	0.13 ± 0.02b	0.12 ± 0.01b	0.12 ± 0.02b	0.14 ± 0.02b	0.23 ± 0.02a
103	unknown-18	-	-	1205/-	0.06 ± 0a	0.04 ± 0.01b	0.03 ± 0b	0.06 ± 0a	0.07 ± 0.01a
104	1,2,3,4-tetramethyl-6-isopropenyl-1,4-Cyclohexadiene	-	*	1213/-	0 ± 0	0 ± 0	0 ± 0	0 ± 0	0.11 ± 0.01
105	β-cyclocitral	432-25-7	**	1214/1216	0.17 ± 0	0.28 ± 0.01	0.29 ± 0.01	0.25 ± 0.03	0.07 ± 0.01
106	6-methyl-1,3-diisopropenylcyclohexene	-	*	1222/-	0.02 ± 0	0 ± 0	0 ± 0	0 ± 0	0.07 ± 0.01
107	unknown-19	-	-	1225/-	0.04 ± 0c	0.05 ± 0.01b	0.05 ± 0b	0.05 ± 0.01b	0.08 ± 0.01a
108	cis-3-hexenyl isovalerate	35154-45-1	*	1230/1235	0.04 ± 0	0.05 ± 0	0 ± 0	0 ± 0	0 ± 0
109	unknown-20	-	-	1234/-	0 ± 0	0 ± 0	0 ± 0	0 ± 0	0.06 ± 0.01
110	2-methyl-2-phenyltridecan	27854-41-7	*	1245/-	0 ± 0	0 ± 0	0 ± 0	0 ± 0	0.05 ± 0
111	unknown-21	-	-	1248/-	0 ± 0	0 ± 0	0 ± 0	0 ± 0	0.03 ± 0
112	unknown-22	-	-	1253/-	0 ± 0	0 ± 0	0 ± 0	0 ± 0	0.07 ± 0.01
113	trans-1-(but-2-en-1-yl)-3,4-dimethylbenzene	54340-86-2	*	1258/-	0 ± 0	0 ± 0	0 ± 0	0 ± 0	0.05 ± 0
114	ionone	8013-90-9	*	1273/-	0.07 ± 0.01d	0.14 ± 0.01c	0.17 ± 0.01b	0.15 ± 0.01b	0.22 ± 0.01a
115	1,3-dimethyl-2-butenylbenzene	50704-01-3	*	1285/-	0 ± 0	0 ± 0	0 ± 0	0 ± 0	0.03 ± 0
116	indole	120-72-9	**	1296/1298	1.38 ± 0.07	0.89 ± 0.12	0.6 ± 0.05	0.46 ± 0.01	0 ± 0
117	tridecane	629-50-5	**	1300/1300	0 ± 0	0 ± 0	0 ± 0	0 ± 0	0.04 ± 0
118	unknown-23	-	-	1308/-	0 ± 0	0.12 ± 0.01	0.1 ± 0.02	0.11 ± 0	0.04 ± 0
119	1,1,6-trimethyl-2H-naphthalene	30364-38-6	*	1346/1358	0.1 ± 0	0.26 ± 0.01	0.33 ± 0.03	0.27 ± 0	0.1 ± 0
120	1,1,6-trimethyl-1,2,3,4-tetrahydro- naphthalene	475-03-6	**	1347/-	0.04 ± 0	0 ± 0	0 ± 0	0 ± 0	0 ± 0
121	unknown-24	-	-	1369/-	0.12 ± 0.02a	0.13 ± 0.01a	0.13 ± 0a	0.12 ± 0.01a	0.1 ± 0b
122	cis-3-hexenyl hexanoate	31501-11-8	**	1378/1382	0.09 ± 0	0.06 ± 0	0.04 ± 0	0.03 ± 0	0.08 ± 0.01
123	β-elemene	515-13-9	*	1383/1406	0.04 ± 0	0.02 ± 0	0.01 ± 0	0.03 ± 0	0.02 ± 0
124	tetradecane	629-59-4	**	1400/1400	0.06 ± 0.01c	0.07 ± 0c	0.11 ± 0.01a	0.09 ± 0.01b	0.07 ± 0.01c
125	(−)-α-cedrene	469-61-4	**	1408/1408	0.01 ± 0	0 ± 0	0 ± 0	0 ± 0	0.02 ± 0
126	unknown-25	-	-	1413/-	0.03 ± 0	0.02 ± 0	0 ± 0	0 ± 0	0.03 ± 0
127	α-ionone	127-41-3	*	1420/1430	0.02 ± 0	0.06 ± 0.01	0.06 ± 0	0.04 ± 0.01	0 ± 0
128	unknown-26	-	-	1425/-	0.03 ± 0c	0.06 ± 0b	0.07 ± 0.01a	0.06 ± 0.01ab	0 ± 0d
129	unknown-27	-	-	1450/-	0.03 ± 0	0 ± 0	0 ± 0	0 ± 0	0.02 ± 0
130	γ-muurolene	30021-74-0	*	1454/1453	0.23 ± 0.01a	0.17 ± 0.01b	0.11 ± 0c	0.11 ± 0.01c	0.03 ± 0d
131	unknown-28	-	-	1461/-	0.02 ± 0	0.03 ± 0	0.01 ± 0.01	0.02 ± 0	0.01 ± 0
132	unknown-29	-	-	1464/-	0.03 ± 0	0 ± 0	0 ± 0	0 ± 0	0.02 ± 0
133	β-ionone	14901-07-6	**	1477/1478	0.06 ± 0.01	0.14 ± 0.01	0.14 ± 0.03	0.18 ± 0.03	0 ± 0
134	α-curcumene	644-30-4	*	1479/1483	0.04 ± 0	0 ± 0	0 ± 0	0 ± 0	0.03 ± 0
135	unknown-30	-	-	1483/-	0.08 ± 0	0 ± 0	0 ± 0	0 ± 0	0 ± 0
136	andrographolide	5508-58-7	*	1490/-	0.16 ± 0.01	0.06 ± 0	0 ± 0	0 ± 0	0.03 ± 0.01
137	γ-himachalene	-	*	1515/1500	1.27 ± 0.07a	0.48 ± 0.03b	0.17 ± 0.01d	0.23 ± 0.04cd	0.28 ± 0.05c
138	unknown-31	-	-	1608/-	0.04 ± 0	0 ± 0	0 ± 0	0 ± 0	0 ± 0
139	(Z)-α-bisabolene	29837-07-8	*	1615/-	0.02 ± 0	0 ± 0	0 ± 0	0 ± 0	0 ± 0
140	unknown-32	-	-	1629/-	2.78 ± 0.1a	1.87 ± 0.09b	1.12 ± 0.07c	0.95 ± 0.08d	0.15 ± 0.04e
141	unknown-33	-	-	1633/-	0.4 ± 0.03	0.09 ± 0.02	0 ± 0	0 ± 0	0.03 ± 0
142	unknown-34	-	-	1651/-	0.03 ± 0b	0 ± 0d	0.04 ± 0a	0.04 ± 0a	0.03 ± 0c
143	unknown-35	-	-	1659/-	0.03 ± 0	0 ± 0	0 ± 0	0 ± 0	0 ± 0

^a^ CAS: the published chemical abstracts service (CAS) of compounds in NIST 11 library; ^b^ MS identification: mass spectrum comparison using NIST 11 library. If the matching degree is greater than 700 (the total value is 1000), and the probability of the first matching compound is 60 higher than the second possible compound, then we defined the first matching compound as absolute identification, marked “**”; if the matching degree is greater than 700, and the probability of the first matching compound is 0–60 higher than the second possible compound, then we defined the result as preliminary identification, marked “*”; if the matching degree is less than 700, so the compound can’t be identified, then we defined it as unknown, marked “-”; ^c^ RI identification: RI_(cal)_ is the measured retention index; RI_(ref)_ is the retention index of the literature; ^d^ ANOVA was applied with *p*-value < 0.05. The same letters indicate that there are no significant difference in the compound’ relative contents between tow tea samples, while different letters indicate that there are significant difference in there. Compounds with no significant difference in relative content among different grades were not labeled.

**Table 2:** Information about 18 different variables.

No.	Compounds ^a^		CAS ^b^	MS ^c^	RI_(cal)_/RI_(ref)_^d^	Peak Area^e^ (Mean ± SD) * 10^6^				
						K110	K101	K102	K103	K104
24	1-ethylpyrrole	+	617-92-5	*	808/-	38.34 ± 3.86a	24.38 ± 0.96b	20.26 ± 2.36c	21.03 ± 0.95bc	10.74 ± 0.77d
140	unknown-32	+	-	-	1629/-	8.67 ± 0.41a	5.25 ± 0.22b	3.28 ± 0.32c	2.83 ± 0.12c	0.98 ± 0.24d
5	2-methylfuran	-	534-22-5	*	-/604	8.84 ± 0.1d	9.39 ± 0.37cd	10.64 ± 0.3b	10.14 ± 0.77bc	22.91 ± 0.79a
12	2-ethylfuran	-	3208-16-0	*	-/701	9.68 ± 0.67c	12.97 ± 1.28b	13.79 ± 1.15b	14.57 ± 1.51b	23.69 ± 2.6a
17	2-methylidenecyclopentan-1-ol	-	20461-31-8	*	-/-	4.38 ± 0.03e	6.27 ± 0.12d	7.1 ± 0.26c	8.07 ± 0.18b	12.99 ± 0.23a
21	mesityl oxide	-	141-79-7	*	-/-	1.91 ± 0.01c	2.13 ± 0.26c	3.51 ± 0.42b	3.56 ± 0.12b	4.66 ± 0.22a
58	2-amylfuran	-	3777-69-3	**	987/986	4.36 ± 0.73d	5.08 ± 0.59cd	6.25 ± 0.95bc	6.81 ± 0.6b	13.88 ± 0.47a
67	D-limonene	-	5989-27-5	**	1025/1025	1.45 ± 0.24c	2.1 ± 0.29c	2.87 ± 0.49b	3.27 ± 0.45b	8.24 ± 0.26a
3	methyl acetate	∨	79-20-9	*	-/-	34.81 ± 0.51b	19.78 ± 1.49c	16.92 ± 1.15d	17.24 ± 1.6d	77.83 ± 0.28a
4	dimethyl sulfide	∨	75-18-3	**	-/-	35.76 ± 1.67b	21.13 ± 2.02c	20.64 ± 0.81c	21.92 ± 1.84c	53.46 ± 0.38a
56	6-methylhept-5-en-2-one	∨	110-93-0	**	983/982	4.93 ± 0.46b	4.59 ± 0.21bc	4.09 ± 0.41c	4.07 ± 0.19c	5.6 ± 0.3a
72	(Z)-β-ocimene	∨	13877-91-3	**	1044/1044	9.45 ± 1.17b	7.58 ± 0.51c	6.78 ± 0.63c	7.18 ± 0.3c	17.72 ± 0.52a
18	toluene	*	108-88-3	**	-/773	7.16 ± 0.38b	6.49 ± 0.66bc	5.65 ± 1c	6.06 ± 0.25bc	37.22 ± 0.99a
10	2-methylbutanal	0	96-17-3	**	-/665	21.14 ± 1.72c	17.13 ± 1.09d	23.13 ± 1.86bc	25.97 ± 1.47a	25.14 ± 0.62ab
11	1-penten-3-ol	0	616-25-1	*	-/681	21.38 ± 1.2d	39.15 ± 1.51c	46.31 ± 1.12a	43.72 ± 1.46b	39.44 ± 0.18c
44	unknown-4	0	-	-	928/-	0.27 ± 0.02bc	0.23 ± 0.02c	0.63 ± 0.01a	0.31 ± 0.03b	0.64 ± 0.05a
86	hotrienol	0	20053-88-7	*	1102/1104	3.52 ± 0.1b	2.69 ± 0.25c	5.46 ± 0.21a	5.41 ± 0.54a	0 ± 0d
91	benzyl cyanide	0	140-29-4	**	1136/1135	4.01 ± 0.28a	3 ± 0.05c	1.45 ± 0.12d	1.53 ± 0.09d	3.61 ± 0.11b

^a^ The relationship between the differential variables and grades: “+” meant positive correlation; “-” meant negative correlation; “∨” meant the peak area decreasing first and then increasing with the decline of grades; “*” meant the compound’ peak area in K104 was significantly higher than that of other grades, but there was negligible difference among other grades; “0” meant negligible difference among different grades; ^b^ CAS: the published chemical abstracts service (CAS) of compounds in NIST 11 library; ^c^ MS identification: mass spectrum comparison using NIST 11 library. If the matching degree is greater than 700 (the total value is 1000), and the probability of the first matching compound is 60 higher than the second possible compound, then we defined the first matching compound as absolute identification, marked “**”; if the matching degree is greater than 700, and the probability of the first matching compound is 0–60 higher than the second possible compound, then we defined the result as preliminary identification, marked “*”; if the matching degree is less than 700, so the compound can’t be identified, then we defined it as unknown, marked “-”; ^d^ RI identification: RI_(cal)_ is the measured retention index; RI_(ref)_ is the retention index of the literature; ^e^ Difference of peak area of differential variables in different grades tea samples. Data were expressed as mean ± SD (standard deviation). ANOVA was applied with *p*-value < 0.05. The same letters indicate that there are no significant differences in the compound peak areas between tow tea samples, while different letters indicate that there are significant difference in there.

**Table 3:** Tea samples used in the study.

Tea Sample Name	Number
special grade of Nongxiang Tieguanyin	K110
first grade of Nongxiang Tieguanyin	K101
second grade of Nongxiang Tieguanyin	K102
third grade of Nongxiang Tieguanyin	K103
fourth grade of Nongxiang Tieguanyin	K104

## Abbreviations

The following abbreviations are used in this manuscript:

HS/GC-MS	Headspace sampling combined with gas chromatography–mass spectrometry
CAS	Chemical abstracts service
MS	Mass spectrometry
SD	Standard deviation
PCA	Principal component analysis
PLS-DA	Partial least squares discrimination analysis
HCA	Hierarchical cluster analysis
VIP	Variable important for the projection
ANOVA	Analysis of variance
